# Design and Implementation of a Multi-Hop Real-Time LoRa Protocol for Dynamic LoRa Networks

**DOI:** 10.3390/s22093518

**Published:** 2022-05-05

**Authors:** Huu Phi Tran, Woo-Sung Jung, Dae-Seung Yoo, Hoon Oh

**Affiliations:** 1 Department of Electrical, Electronic and Computer Engineering, University of Ulsan, Ulsan 680-749, Korea; huuphi.tran88@gmail.com; 2 Electronics and Telecommunications Research Institute, Daejeon 305-350, Korea; woosung@etri.re.kr (W.-S.J.); ooseyds@etri.re.kr (D.-S.Y.)

**Keywords:** multi-hop LoRa protocol, dynamic network, mobility, real-time scheduling, reliable transmission

## Abstract

Recently, LoRa (Long Range) technology has been drawing attention in various applications due to its long communication range and high link reliability. However, in industrial environments, these advantages are often compromised by factors such as node mobility, signal attenuation due to various obstacles, and link instability due to external signal interference. In this paper, we propose a new multi-hop LoRa protocol that can provide high reliability for data transmission by overcoming those factors in dynamic LoRa networks. This study extends the previously proposed two-hop real-time LoRa (Two-Hop RT-LoRa) protocol to address technical aspects of dynamic multi-hop networks, such as automatic configuration of multi-hop LoRa networks, dynamic topology management, and updating of real-time slot schedules. It is shown by simulation that the proposed protocol achieves high reliability of over 97% for mobile nodes and generates low control overhead in topology management and schedule updates. The protocol was also evaluated in various campus deployment scenarios. According to experiments, it could achieve high packet delivery rates of over 97% and 95%, respectively, for 1-hop nodes and 2-hop nodes against node mobility.

## 1. Introduction

Recently, interest in LoRa networks has been increasing due to the absence of an industrial IoT network capable of reliable data transmission at a low price. In keeping with this need, remarkable research progress has been made on industrial multi-hop LoRa networks by enabling reliable real-time data transmission and supporting a wide area with many nodes with the use of a slot scheduling-based data transmission method [1]. However, in industrial applications supporting worker safety, real-time machine management, etc., LoRa terminals (nodes) can be attached to mobile equipment or carried by workers. This requires a data transmission protocol for dynamic multi-hop LoRa networks. However, designing a reliable and real-time data transmission protocol in a dynamic multi-hop LoRa network is a challenge because it involves topology maintenance in a slot scheduling-based approach. This problem gets worse in LoRa networks with a low data rate and a high probability of message collisions.

So far, many studies designed to deal with reliable data transmission in dynamic wireless networks have been conducted under three areas of communication technology: cellular networks, wireless sensor networks (WSNs), and low-power wide-area networks (LPWANs). The cellular network has the advantage of reliably collecting data while covering a wide area and can easily support node mobility because it uses a simple star topology and operates at high bandwidth. However, when a node enters a wireless communication shadow area, it suffers from reduced reliability of data transmission and high energy consumption. Moreover, cellular networks are seldom used for monitoring applications except for special cases because every node has to pay an expensive communication fee.

Meanwhile, there have been many studies on industrial WSNs based on the IEEE 802.15.4 standard [2] for low-cost data collection over the past decade. In WSNs, nodes that are typically considered stationary send data to a sink via multiple radio hops. Several protocols using a slot scheduling have been proposed to transmit data reliably in WSNs [3,4,5,6]. However, in industrial sites, even though nodes send data according to a slot schedule, the reliability of data transmission may not be secured due to various communication obstacles and external interferences. Furthermore, node mobility not only increases control overhead but also causes data loss temporarily due to link failure and frequent updates of a slot schedule, making it more difficult to secure data transmission reliability. Recently, the slotted sense multiple access (SSMA) protocol [7] using a tree topology and sharable slots addressed the issue of data transmission reliability under various signal interferences in industrial environments and node mobility. In this method, instead of allocating a tiny slot to each link, a shareable slot is allocated to each tree level of the tree topology, and nodes at each tree level send data to nodes one tree level below them through contention using CSMA/CA within a sharable slot allocated to their tree level. In this way, data transmission is progressively performed level by level from the highest tree level to the lowest tree level to which a sink node belongs. This method eliminates scheduling overhead by making slot scheduling topology-independent and greatly improves the reliability of data transmission by limiting channel contention to nodes in the same tree level. However, since this approach still allows channel contention, it is not free from data collision. To further improve the reliability, the authors in [8] proposed a smart multi-channel *SSMA* (SMC-SSMA) protocol. In this approach, in the process of acquiring a common channel for secure data transmission, each node includes “delay time before sending its own control message” in the control message it transmits. Then, every node learns the delay times of its neighboring nodes and hidden nodes by overhearing the control messages and then sets its own delay time before sending a control message so that its control messages can never collide with those transmitted by its neighboring and hidden nodes. After acquiring the common channel, a node transmits data using a data channel not used by its neighbors, enabling parallel transmission.

Despite remarkable progress in these dynamic WSNs, WSNs in industrial fields still have difficulties in securing data transmission reliability due to obstacles and/or external interference and have limitations in covering a wide area or supporting densely deployed nodes. Some studies [9,10,11,12,13,14] on the use of LoRa technology [15] to overcome those difficulties and limitations have been conducted. It is said in the LoRa specification [16] that a LoRa transceiver can cover up to 2 km even with the smallest spreading factor; however, its transmission range can be reduced to within hundreds or tens of meters due to signal attenuation by obstructions and the installations of nodes inside *wireless unfriendly zones* (WUZs) such as underground tunnels and enclosed spaces. Furthermore, in industrial monitoring and control applications, a server may collect data from each node every tens of seconds or even every few seconds. Therefore, a LoRa protocol must address how to achieve high reliability of data transmission against signal attenuation and heavy traffic.

Some LoRa protocols have been proposed to tackle the problem of reliability and/or network coverage. In [13], the authors proposed the real-time LoRa (RT-LoRa) protocol that enables real-time and reliable data transmission by using a distributed slot scheduling method. Another slot scheduling-based protocol, TS-LoRa [14], allows nodes to determine a slot in a frame autonomously in order to reduce slot scheduling overhead. However, these protocols suffer from the limitation of network coverage in industrial fields. To resolve coverage limitation, some studies focused on multi-hop LoRa networks [17,18,19,20,21,22]. In [17], the protocol extends network coverage by having the end node transmit data using a route established by a simplified Destination-Sequenced Distance Vector (DSDV) protocol [23]. This protocol suffers from high energy consumption by routing data via multiple radio hops, and also from data collision due to the nature of contention-based data transmission in high traffic. The authors in [18] proposed CT-LoRa, a multi-hop LoRa protocol based on the Glossy protocol [24], that takes advantage of *concurrent transmission* (CT) to improve network reliability. This approach relies on flooding for data transmission while removing the overhead of constructing and maintaining a multi-hop topology. However, the flooding is not free from the viewpoint of network overhead and energy consumption. The studies of the same category include the LoRa-Mesh protocol [19] in which a GW constructs and maintains a tree path to every node in the network by selecting a path that has the smallest hop count and provides a good link quality. In order to get data from a specified node, GW sends a query message to the node along the tree path. This approach improves the reliability of data transmission significantly; it incurs a high control overhead. According to experiment results, the above-mentioned multi-hop LoRa protocols effectively extend the network coverage as well as provide high reliability on data transmissions. However, these protocols may not be suitable for monitoring applications because they have difficulty in dealing with high traffic networks.

Recently, the authors in [1] proposed the Two-Hop RT-LoRa protocol to extend network coverage in which nodes send data periodically, while some nodes make use of relay nodes to send data to GW. The protocol uses a real-time slot schedule for a two-hop tree topology to remove data collision while it satisfies the time constraint of every data transmission. According to the experimental results, the two-hop protocol using the lowest spreading factor SF7 showed a high packet delivery rate of more than 95% in the rough network deployment scenario where the one-hop protocol using the relatively high spreading factor SF10 mostly fails to transmit data. In addition, according to the analysis results, the two-hop protocol could improve energy consumption by almost 60% compared to the one-hop protocol. However, the protocol did not deal with the change of topology. In wireless networks with a relatively high bandwidth such as WiFi and IEEE 802.15.4, the change of topology may be easily handled by exchanging control messages. However, in a dynamic LoRa network, a method is required to quickly detect link failures and update slot schedules efficiently in response to topology changes while using a small number of control messages to reduce message collisions. To the best of our knowledge, no one has ever conducted research on a multi-hop LoRa protocol that can deal with node mobility.

This paper presents a reliable and real-time data transmission protocol for dynamic multi-hop LoRa networks. This study basically extends the slot scheduling-based Two-Hop RT-LoRa protocol proposed in the previous study [1], further covering the technical aspects of the implementation and operation of a dynamic multi-hop LoRa network such as auto-configuration of a multi-hop LoRa network, slot scheduling, topology maintenance, and updating of a slot schedule. First, in autonomously configuring a two-hop network, it is important to ensure that the tree topology can be gradually and stably built in the process of individual node registration. Therefore, a GW includes the list of registered nodes in the tree construction message before broadcasting so that a node can confirm whether it is registered or not. Considering that the one-hop relay nodes play an important role in the stability of tree topology, a node decides whether it can become a relay by itself based on its link quality. As a server generates and broadcasts slot scheduling information based on the tree topology, each node can easily and autonomously create a slot schedule that does not conflict with the slot schedules of other nodes. In this process, the server provides the retransmission slot schedule for relay nodes so that all relay nodes can safely rebroadcast the slot scheduling information to their children without collision. During data transmission, when a node detects link failure, it immediately reports the change of link state to the GW using an unscheduled slot to prevent collision with other data transmissions. Upon receiving this, the GW completes topology maintenance by transmitting only updated slot scheduling information using the DL message.

The rest of this paper is organized as follows. Section 2 describes the research background. Section 3 gives a detailed design of the proposed protocol and is followed by the discussion of simulation and experimental results in Section 4. Finally, the conclusion is drawn in Section 5.

## 2. Background

### 2.1. Network Model

A considered LoRa network consists of one server, multiple *gateways* (GWs) and a number of *end devices* or *nodes*. A server and GWs are interconnected by a wired high-speed backhaul network. End nodes are mobile and battery-powered. A server (or a GW) collects sensor data from end nodes via GWs periodically and provides services based on the analysis of the collected data. Nodes may be installed in a WUZ, such as an underground tunnel and an enclosed space. Some nodes may not have a direct connection to the GW due to signal attenuation. Every node can act as a relay that forwards data to a GW. Nodes can form a two-hop tree originating from a GW in which an internal node acts as a *relay node*, and a leaf node can be either a *1-hop node* that connects to GW directly or a *2-hop node* that connects to a relay node. A node that does not belong to a tree is said to be an *orphan node*. For time synchronization and/or command transmission, a GW broadcasts a downlink message periodically to all end nodes. Thus, all relay nodes are to rebroadcast the received downlink message towards 2-hop nodes.

**Figure 1:**
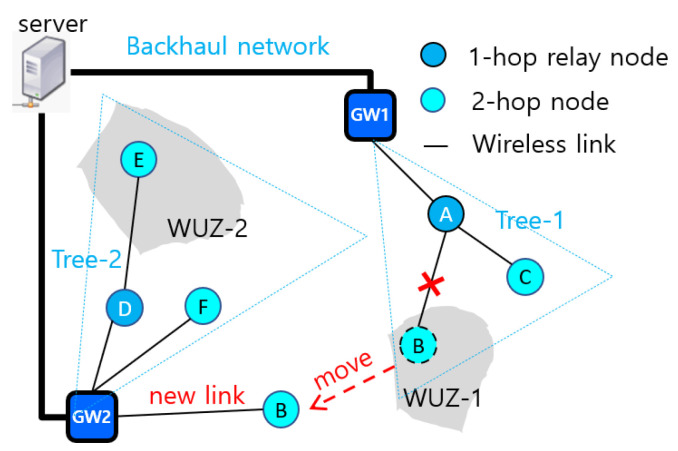
A LoRa network model with two-hop tree topology.

Figure 1 shows a simple LoRa network with two GWs and six end nodes. Two nodes, *B* and *E*, are deployed in WUZ-1 and WUZ-2, respectively. Nodes *A*, *B*, *C* form *Tree-1* originated from GW1, and nodes *D*, *E*, *F* form *Tree-2* originated from GW2 where nodes *A* and *D* are relay nodes. The figure also illustrates the movement of node *B* connecting to GW2 after disconnecting from node *A*.

### 2.2. Problem Identification

Recently, the authors in [1] proposed a Two-Hop Real-Time LoRa protocol that uses a two-hop tree topology for extension of network coverage and a two-hop slot scheduling for reliable data transmission. Based on the slot scheduling information transmitted by a GW, every node generates its own slot schedule in a distributed manner such that it satisfies its own transmission period if it transmits data according to the slot schedule, and does not cause any collision in data transmission with other nodes. However, this approach does not respond to the change of topology.

**Figure 2:**
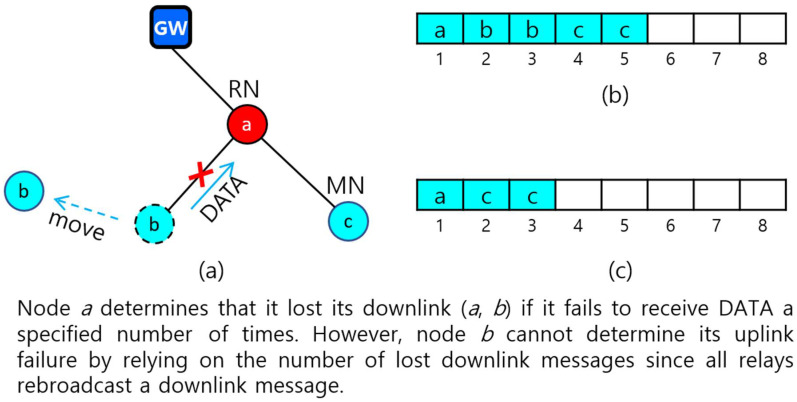
Link failure and inconsistency of slot schedule due to node mobility. (**a**) The change of topology by node movement; (**b**) Slot schedule before node *b* moves away; (**c**) Slot schedule after node *b* moves away.

There are some issues to consider in designing a real-time protocol for dynamic two-hop LoRa networks. First, the protocol should be able to detect the uplink or downlink failure of a node quickly. A node, either a GW or a relay node, can detect its downlink failure by counting the amount of missing data from its child. Furthermore, a 1-hop (relay) node can detect its uplink failure easily by counting the number of missing downlink messages from a GW. However, it is not possible for a 2-hop node to use a downlink message to detect uplink failure. The reason is that all relay nodes rebroadcast an identical downlink message. Therefore, this requires a different method using downlink messages. One way is that a 2-hop node can detect its uplink failure by using the downlink failure information of its parent. For example, consider a simple two-hop network topology in Figure 2a that consists of relay node *a* and 2-hop nodes *b* and *c*. Let a downlink state of node *x* with *k* children, $x_{1},x_{2},\ldots ,\;\mathrm{and}\;x_{k}$, be represented as $(x(x_{1},x_{2},\ldots ,\;x_{k}$)). Suppose that node *a* detected the failure of its downlink (*a*, *b*) and reported its changed downlink state (*a*(*c*)) to GW. If the GW broadcasts (*a*(*c*)), node *b* will know of the failure of uplink (*b*, *a*) if it has already connected to GW.

Second, the protocol should be able to update a slot schedule quickly for the change of topology. If GW detects the downlink failure of a 1-hop relay or leaf node, the GW simply releases the slots allocated to that node and its children, and then broadcasts a downlink message to request the relevant nodes to switch to orphan nodes. Then, each orphan node needs to individually rejoin the tree and be allocated slots. However, when a relay node detects a downlink failure for a child, the slot rescheduling becomes a bit more complicated. In this case, the relay node will first report its changed downlink state to GW so that the GW can generate a new slot schedule for the relay node. The child can know its uplink failure if it receives the changed link state of its parent from GW. For example, referring to Figure 2, suppose that GW has a *slot schedule* (SS) for the downlink state (*a*(*b*, *c*)) of node *a*, as *SS*(*a*) = (*a* = (1), *b* = (2, 3), *c* = (4, 5)) as shown in Figure 2b. Note that every 2-hop node need two slots, one for its own transmission and another for its parent to forward the received data. If node *a* detects its downlink failure due to the movement of node *b* and reports a changed downlink state (*a*(*c*)) to GW, the new slot schedule becomes *SS*(*a*) = (*a* = (1), *c* = (2, 3)) as in Figure 2c.

Third, the protocol has to handle a *slot schedule conflict problem* that occurs when multiple nodes use the same slot temporarily. Suppose that node *a* has a new slot schedule shown in Figure 2c after it detects the failure of its downlink (*a*, *b*). The problem is that node *b* may still use its previous slot number 2 until it finds a new parent.

Fourth, as GWs and relay nodes form the mobile backbone of the LoRa network, it is of great importance to build and maintain the network topology in such a way that the relay nodes have stable uplink. If a one-hop relay node loses an uplink, a significant amount of overhead may occur in the process of topology change and slot rescheduling.

Finally, one important question is whether it is or is not appropriate to have three or more radio hops in a low data rate LoRa network, even at the cost of the increased control overhead and the increased likelihood of collisions due to increased traffic. For example, if *k-hop* is allowed in multi-hop LoRa networks, the data generated at the (*k* + 1)^th^ tree level must be transmitted *k* times before reaching a GW. This may increase interference severely due to the long transmission distance of LoRa. However, even though *k* is limited to 2, the use of two GWs can extend the coverage of a LoRa network up to eight hops such that two GWs cover three nodes arranged linearly between them, and each GW additionally covers two nodes arranged linearly on opposite sides as $(x_{1}-x_{2}-G_{1}-x_{3}-x_{4}-x_{5}-G_{2}-x_{6}-x_{7}$) where $G_{1}$ and $G_{2}$ are gateways and $x_{i},\;i=1\ldots 7$, indicates an end node.

In conclusion, this paper aims at designing a multi-hop LoRa protocol in consideration of the issues discussed so far.

### 2.3. Notations and Messages

A node can be modelled as a task, an active entity that receives command and transmits data. In this paper, we assume that each node has only one task. Thus, task and node are used interchangeably. A task belongs to a specific task class based on its data transmission interval (TI) such that for a frame with 2*^N^* uplink slots, where *N* is defined as a frame factor (*N* ≥ 0), a task belongs to class *c* (0 ≤ *c* ≤ *N*) if it transmits one data per *TI* = 2*^N^*/2*^c^*. This implies that a task of class *c* has a slot demand (*SD*) of 2^*c*^ slots and transmits 2*^c^* packets during one frame period. Task *x* can be expressed by its profile *PF*(*x*) as follows:

(1) PF(x)=(x,class(x))

where *x* and *class(x)* indicate node address and the class of node *x*, respectively.

Some notations used in this paper are summarized as follows:
NotationMeaning*RNL*indicates a *registered node list* in which every node has registered with a server.*P(x)*indicates the parent of node *x*.*TCRInt*indicates the interval that GW uses to broadcast a *tree construction request* (*TCR*) message during initialization period.*MaxChildren*indicates the *maximum number of children* that a 1-hop relay node can have.*uPF(x*indicates the *updated profile* that node *x* generates if it detects link breakage to any of its children.*uSSI(x)*indicates an *updated slot scheduling information* that a server generates for node *x* that has reported *uPF(x)*

Some messages used in this paper are summarized as follows:
MessageDescription*TCR* = (*level*, *RNL*)is a *tree construction request (TCR)* message that a server broadcasts at the intervals of *TCRInt* during initialization period and *level* indicates the tree level of the node that broadcasts this message.*RR(x) = (x,P(x),PF(x))*is the *registration request (RR)* message that node *x* sends to its parent *P(x)* to register with a server.

### 2.4. Overview of Slot Scheduling for Two-Hop LoRa Networks

The Two-hop RT-LoRa protocol uses a frame as a data collection cycle that is divided into a *downlink* (DL) period and an *uplink* (UL) period that GW uses to broadcast a DL message and end nodes use to send data, respectively. The DL period is further divided into two DL slots: DL#1 for GW to broadcast a DL message and DL#2 for 1-hop relay nodes to rebroadcast the DL message, and the UL period is sliced into 2^*N*^ data slots.

**Figure 3:**
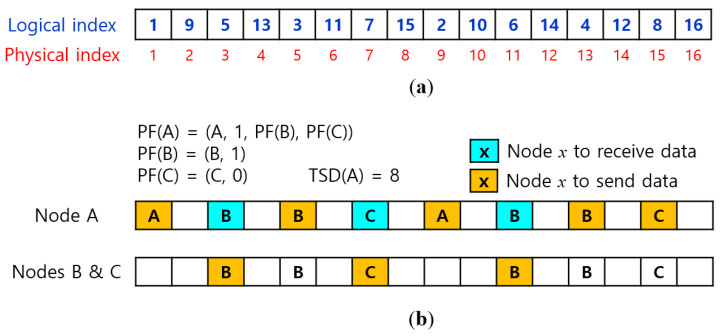
Two-hop tree slot scheduling using logical slot indices. (**a**) Logical slot indexing with 16 slots; (**b**) Slot scheduling for Tree-1 in Figure 1.

Given a set of tasks, the protocol performs slot scheduling by relying on the *logical slot indexing* (LSI) algorithm [13], that assigns a logical slot index to each of 2*^N^* data slots such that if a task of class *c* selects 2*^c^* logical slots sequentially starting with any logical slot index and transmits data in each logical slot, it can meet the transmission period of 2*^N^/2^c^*. The logical slot indices for 16 data slots are given in Figure 3a.

For slot scheduling, every node is required to report its profile to a server. A 1-hop relay collects the profiles of its children and merges them with its own task profile before reporting. Suppose that a network has a list of *k* 1-hop nodes as (*n*_1_, *n*_2_, …, *n_i_*, …, *n_k_*). Then, the integrated profile *PF*(*n_i_*) is expressed as follows:

(2) $$PF(n_{i})=(n_{i},class(n_{i}),PF(n_{i1}),PF(n_{i2}),\ldots ,PF(n_{ij}),\ldots ,PF(n_{ic_{i}}))$$

where *PF*(*n_ij_*) indicates the profile of the *j*^th^ child of node *n_i_*, and *c_i_* is the number of *n_i_*’s children. Let *SD*(*x*) and *TSD*(*x*) denote the slot demand of node *x* and the total slot demand of *x* and *x*’s children, respectively. Then, *TSD*(*n_i_*) is expressed as follows:

(3) $$TSD(n_{i})=SD(n_{i})+\sum_{j=1}^{c_{i}}SD(n_{ij})$$

where $SD(n_{i})=2^{class(n_{i})}$ and $SD(n_{ij})=2*2^{class(n_{ij})}$ since 2-hop node needs twice as many slots as it demands. Then, every 1-hop node *n_i_* reports *PF*(*n_i_*) to a server so that the server can manage the task profile (*PF*) for all nodes in the network as follows:

(4) $$PF=(PF(n_{1}),PF(n_{2}),\ldots ,PF(n_{k}))$$

If tasks are scheduled in the order of the elements in *PF*, the start logical slot index of node *n_i_*, *startLSI*(*n_i_*), in slot schedule is calculated as follows:

(5) $$startLSI(n_{i})=\sum_{j=1}^{i-1}TSD(n_{j})+1$$

A sever calculates *total slot demands* for all 1-hop nodes according to (3), and distributes the *network slot scheduling information* (*NSSI*) using a DL message:

(6) $$NSSI=((n_{1},TSD(n_{1})),(n_{2},TSD(n_{2})),\ldots ,(n_{k},TSD(n_{k})))$$

Upon receiving *NSSI*, every 1-hop node *x* gets its *startLSI*(*x*) according to (5) and generates a *local slot schedule*, *LSS*(*x*), using Algorithm 1 with *startLSI*(*x*) and *TSD*(*x*). The *LSS*(*x*) consists of its *receiving slots*, *RxSlots*(*x*), used to receive data from its children and its *transmitting slots, TxSlots*(*x*), used to transmit its own data and relay data received from its children.
**Algorithm 1.** Slot scheduling of 1-hop relay node1:  At node *x* that receives *NSSI*: 2:    calculates *startLSI*(*x*) using (5); 3:    *Alloc*(*x*) = a list of *SD*(*x*) sequential logical slot numbers starting with *startLSI*(*x*) according to the LSI algorithm; //The ascending sorted list of physical slot numbers corresponding to *Alloc*(*x*) 4:    *TxSlots*(*x*) = ascSort {psi(*y*)| *y* ∈ *Alloc*(*x*)}; 5:        *startLSI* = *startLSI*(*x*) + *SD*(*x*); 6:    *RxSlots*(*x*) = { }; 7:    **for each** *y* ∈ CS(*x*) 8:       *Alloc*(*y*) = a list of *SD*(*y*) sequential logical slot numbers starting with *startLSI*; 9:       psiAlloc(y) = ascSort {psi(*v*)| *v* ∈ *Alloc*(*y*)} 10:       *RxSlots*(*x*) = *RxSlots*(*x*) ∪ {*v*| *v* ∈ psiAlloc(*y*), *v* is in odd position}; 11:       *TxSlots*(*x*) = *TxSlots*(*x*) ∪ {*v*| *v* ∈ psiAlloc(*y*), *v* is in even position}; 12:       *startLSI* = *startLSI* + *SD*(*y*); 13:    **endFor**

Furthermore, 1-hop relay node *x* generates its *local slot scheduling information*, *LSSI*(*x*), that is required for its children to perform slot scheduling:

(7) $$LSSI(x)=(startLSI,PF(x_{1}),PF(x_{2}),\ldots ,PF(x_{k_{x}}))$$

where *startLSI* = *startLSI*(*x*) + *SD*(*x*), and *k_x_* indicates the number of node *x*’s children. Then, node *x* broadcasts *LSSI*(*x*), and its child generates a slot schedule that includes *TxSlots* using Algorithm 2.
**Algorithm 2.** Slot scheduling of 2-hop node1:  At node *x_i_* that receives *LSSI*(*x*): 2:    $startLSI=startLSI+\sum_{j=1}^{i-1}SD(x_{j});$ 3:    get *Alloc*(*x_i_*) starting with *startLSI*; 4:    psiAlloc(*x_i_*) = ascSort {psi(*v*)| *v* ∈ *Alloc*(*x_i_*)}; 5:    *TxSlots*(*x_i_*) = {*x*| *x* ∈ psiAlloc(*x_i_*), *x* is in odd position};

Let us give an example to generate *LSS*(*A*) in Figure 1. Suppose that *PF*(*A*) = (*A*, 1, (*B*, 1), (*C*, 0)) and *startLSI*(*A*) = 1. Then, *TSD*(*A*) = 8 and *Alloc*(*A*) = (1, 2), *Alloc*(*B*) = (3, 4, 5, 6), and *Alloc*(*C*) = (7, 8). Then, we get the ascending-sorted physical slot indices: psiAlloc(*A*) = (1, 9), psiAlloc(*B*) = (3, 5, 11, 13), and psiAlloc(*C*) = (7, 15). The underlined numbers in even positions are transmission slot numbers: *TxSlots*(*A*) = (1, 5, 9, 13, 15), and *RxSlots*(*A*) = (3, 7, 11) that corresponds to *TxSlots*(*B*) ∪ *TxSlots*(*C*) from Algorithm 2. The slot schedule is illustrated in Figure 3b.

## 3. Protocol Design

### 3.1. Protocol Structure

**Figure 4:**
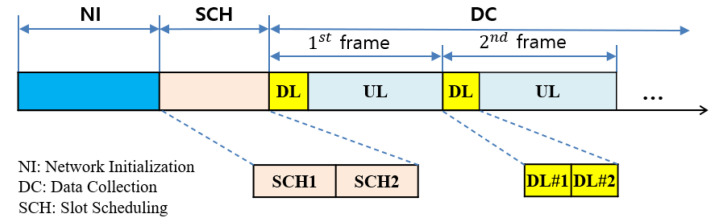
The operational structure of the proposed protocol.

The protocol operation starts with *network initialization* (NI). As each node is installed, it starts registering with a server via GWs immediately, and the installed nodes progressively form a two-hop tree originating from a GW. When a specified percentage of end nodes are registered, the server starts a *slot scheduling* (SCH) period. Unregistered nodes are registered later during the *data collection* (DC) period. The SCH period is divided into two subperiods, SCH1 and SCH2, for the slot scheduling of 1-hop nodes and that of 2-hop nodes, respectively. Then, the server initiates a data collection period that repeats a *frame* or *data collection cycle*. The maintenance of network topology is made continuously during data collection. The operational structure of the proposed protocol for dynamic LoRa networks is illustrated in Figure 4.

### 3.2. Frame-Slot Architecture

With *m* channels, *m* overlapping frames can be defined during one frame period. A frame using channel *Ch_i_* is denoted by *Ch_i_-frame*. A GW broadcasts a DL message during DL#1, and 1-hop relay node rebroadcasts the received DL message during DL#2 while all nodes listen to the common channel. Upon receiving the DL message, every node synchronizes the start time of the UL period.

**Figure 5:**
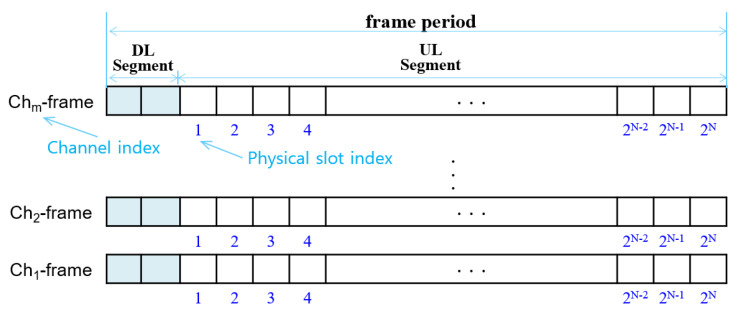
Multi-channel frame-slot architecture.

The UL period is further sliced into 2*^N^* data slots where *N* as a *frame factor* is an integer constant, and each data slot is sufficiently large to send one data packet. The data slots in the UL period are identified by *physical slot indices*, numbered sequentially from 1 to 2*^N^*. A frame-slot architecture using *m* channels is illustrated in Figure 5.

### 3.3. Network Initialization

During the NI period, nodes register with a server and form a two-hop tree topology by considering link quality. A server starts constructing a tree by having a GW broadcast a *tree construction request* (*TCR*) message at regular intervals. The *TCR* includes a current *registered node list* (*RNL*), which is empty at the beginning and is updated whenever a server receives a *registration request* (*RR*) message from a new node.

**Figure 6:**
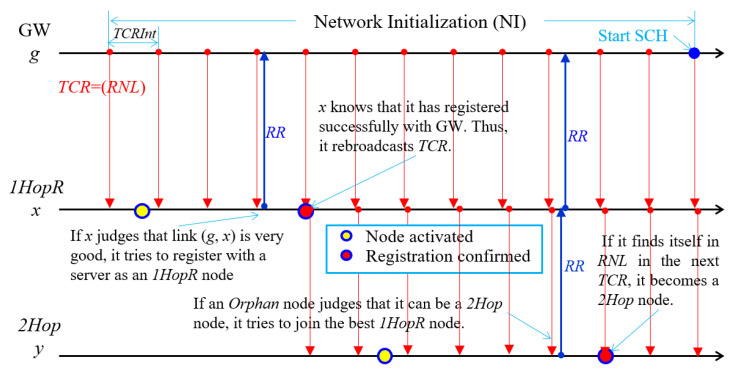
Node registration and tree construction process.

A node determines its *node type* (*nodeType*) using a *received signal strength indicator* (*RSSI*) and a *signal-to-noise ratio* (*SNR*) after receiving multiple *TCR*s and comparing them with the specified threshold values, *RSSI_Th1*, *RSSI_Th2*, *SNR_Th1*, and *SNR_Th2* such that *RSSI_Th1* > *RSSI_Th2* and *SNR_Th1* > *SNR_Th2*. An orphan (*Orphan*) node turns into a 1-hop (*1Hop*) node or a 1-hop relay (*1HopR*) node if the uplink quality is good; otherwise, it remains a 2-hop candidate (*2HopCan*) node temporarily, as detailed in Algorithm 3. If a *2HopCan* node determines its parent, it becomes a 2-hop (*2Hop*) node. Since a *1HopR* node determines the stability of the network, its uplink has to be highly robust.
**Algorithm 3.** Node type determination//*avg_rssi* indicates the average of *RSSI*s //*avr_snr* indicates the average of *SNR*s 1:  At an *Orphan* node that receives *k TCR*s, *k* > 1: 2:    calculate *avg_rssi* and *avg_snr* with multiple *TCR*s; 3:    determine *nodeType* using *avg_rssi* and *avg_snr* as follows: 4:    **if** *avg_rssi* ≥ *RSSI_Th1* and *avg_snr* ≥ *SNR_Th1* **then** 5:       *nodeType* = *1HopR*; 6:    **else if** *avg_rssi* ≥ *RSSI_Th2* and *avg_snr* ≥ *RSSI_Th2*
**then** 7:       *nodeType* = *1Hop*; 8:    **else** 9:       *nodeType* = *2HopCan*;

A *2HopCan* node turns to a *2Hop* node only if it can join any *1HopR* node. The joining process is as follows. A *2HopCan* node waits to receive more *TCR*s to find a good *1HopR* node. Suppose that it received *TCR*s from multiple *1HopR* nodes. Then, it first selects all *1HopR* nodes with *RSSI* and *SNR* greater than and equal to *RSSI_Th2* and *SNR_Th2*, respectively. Then, a *2HopCan* node turns to a *2Hop* node if it can connect to any *2HopR* node with the minimum link quality that can maintain connectivity. This is quite reasonable in LoRa networks since the data transmitted from any *2Hop* node can reach GW directly or indirectly via *1HopR* node, thereby increasing the probability of data delivery to GW. The effect of increasing the reliability of transmission due to data being transmitted additionally along another path without wasting spectrum is referred to as an augmented transmission effect. The *2HopCan* node *x* selects a *1HopR* node with the largest RSSI value as a candidate parent, say *y*, among the selected *1HopR* nodes to send *RR*. Upon receiving *RR*, node *y* forwards *RR* to GW only if it has children less than or equal to *MaxChidren*. In this process, since node *x* can register with the server without *1HopR* node *y*’s knowledge, the GW must discard the *RR* sent directly by node *x*. When node *x* finds itself in *RNL* the next time it receives *TCR*, it becomes a *2Hop* node. Otherwise, node *x* must hold off joining the network until the start of the DC period. Tree construction and node registration process of a *1HopR* node *x* and a *2Hop* node *y* with GW g is illustrated in Figure 6.

In this process, every node, say *x*, maintains a node information table, *NodeIT*(*x*), as follows:

(8) NodeIT(x)=(P(x),level(x),RSSI(P(x)),SNR(P(x)),CS(x))

where *P*(*x*) is the parent of node *x*, *level*(*x*) is the tree level of node *x*, *RSSI*(*P*(*x*)) and *SNR*(*P*(*x*)) indicate *RSSI* and *SNR* for a link (*x*, *P*(*x*)), respectively, and *CS*(*x*) indicates the set of node *x*’s children.

If a server finds a specified portion of nodes registered, it initiates SCH period by broadcasting slot scheduling information.

### 3.4. Scheduling Information Dissemination and Slot Scheduling

During the NI period, a server manages the full tree topology and the profile of all nodes with the received *RR*s. For the convenience of management, it divides all nodes into *m* groups corresponding to *m* channels and distributes scheduling information in groups.

Suppose that group *i* has a list of *k_i_* 1-hop nodes as $\left(n_{i1},n_{i2},\ldots ,n_{ik_{i}}\right)$. Then, a server generates *group slot scheduling information*, *GSSI(i*) for group *i* as follows:

(9) $$GSSI(i)=(i,(n_{i1},TSD(n_{i1})),(n_{i2},TSD(n_{i2})),\ldots ,(n_{ik_{i}},TSD(n_{ik_{i}})))$$

where $k_{i}$ is the number of 1-hop nodes in group *i*, and $n_{ij}$ indicates the *j*^th^ 1-hop node. Then, the network slot scheduling information (*NSSI*) can be represented in terms of groups as follows:

(10) NSSI=(GSSI(1),GSSI(2),…,GSSI(m))

Then, the server broadcasts the entire *NSSI* to all 1-hop nodes by broadcasting *slot scheduling information* (*SSI*) messages *m* times in such a way that it broadcasts the *SSI*, including *GSSI*(*i*) in the *i*^th^ slot among the *m* slots of SCH1. If the size of the *SSI* is too large, the server segments it and transmits each successively. Then, each *1HopR* node, *x*, generates *LSSI*(*x*) as follows:

(11) $$LSSI(x)=(g(x),startLSI,PF(x_{1}),PF(x_{2}),\ldots ,PF(x_{k_{x}}))$$

where *g*(*x*) indicates the group to which node *x* belongs, and *startLSI = startLSI(x) + SD(x)*, and $k_{x}$ indicates the number of children of node *x*. This implies that node *x* obtains its slot demand starting at *startLSI*(*x*) and its children obtain their slot demands starting at *startLSI* in the frame, *Ch_g(x)_-frame*.

**Figure 7:**
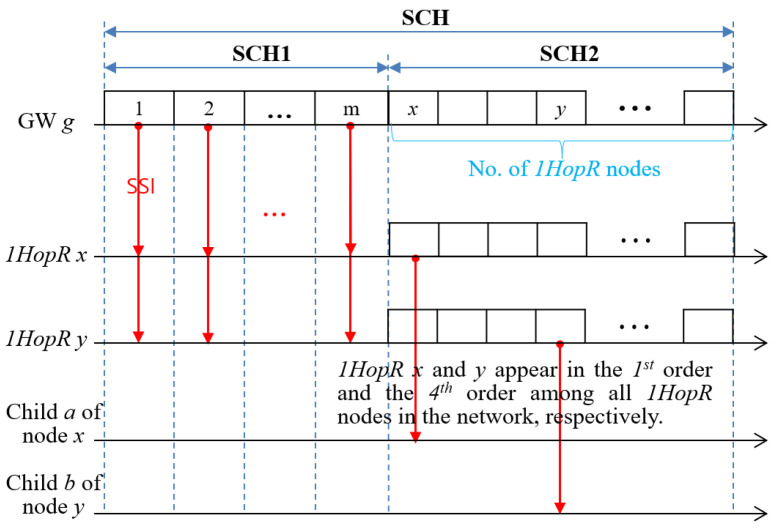
Distribution of group scheduling information.

To prevent collision during the distribution of *LSSI*, every *1HopR* node *x* broadcasts *SSI* = (*LSSI*(*x*)) in the *i*^th^ slot of the SCH2 period if it appears in the *i*^th^ order among *1HopR* nodes belonging to the *NSSI* as shown in Figure 7. The slot scheduling for *1HopR* and *2Hop* nodes follows Algorithm 1 and Algorithm 2, respectively.

### 3.5. Data Transmission and Topology Maintenance

Every node transmits data to its parent according to the slot schedule while a server broadcasts a DL message at the beginning of every frame. This section describes the methods to deal with link failure against node mobility, node joining and leaving, and the update of a slot schedule during data transmission.

#### 3.5.1. Link Failure

A link has a time-varying condition due to node movement, signal interference, or the intervention of obstacles. Thus, a node and its parent should be able to detect and repair link failure and update a slot schedule accordingly.

A node detects downlink failure by making use of data transmission. If node *x* has not received data from any child *y* for three consecutive frames, it decides that link (*x*, *y*) is broken and updates *NodeIT*(*x*) with *CS*(*x*) = *CS*(*x*) − *y*. If node *x* is GW, it releases the slots allocated to node *y*. If node *x* is of *1HopR*, it creates an updated profile *uPF*(*x*) for its new link state as follows:

(12) uPF(x)=(x,class(x),{PF(c)|c∈CS(x)})

Then, *1HopR* node *x* sends *RR* = (*x*, *P*(*x*), *uPF*(*x*)) to GW.

To avoid collision, a node transmits *RR* using an unscheduled slot in its frame. Upon receiving *RR*, a server generates an updated slot scheduling information, *uSSI*(*x*), for *1HopR* node *x* as follows:

(13) uSSI(x)=(g(x),startLSI(x),uPF(x))

Then, the server includes *uSSI*(*x*) in the next *DL* message so that node *x* and node *x*’s children can update their slot schedules.

However, it is not possible for a 2-hop node to detect that the uplink to its parent is down using a DL message. The reason is that since all *1HopR* nodes broadcast the same DL message at the same time, the node does not always receive a DL message its parent node broadcasts. In fact, a *2Hop* node can receive a DL message through various routes, such as a GW, its parent *1HopR* node, or other *1HopR* nodes. Therefore, very conservatively, if a node does not receive a DL message for three consecutive frame periods, it determines that the uplink is down and changes its type to *Orphan*. An additional way for *2Hop* node *x* to detect uplink failure is to analyze *uSSI*(*P*(*x*)) in the DL message. If *2Hop* node *x* finds itself removed from *uSSI*(*P*(*x*)), it decides that its uplink (*x*, *P*(*x*)) is down and changes its type to *Orphan*.

#### 3.5.2. Node Joining

To maintain the reliability of data transmission, an *Orphan* node should be able to join a GW or *1HopR* node without interfering with other data transmissions. Therefore, every *1HopR* node *r* always includes two parameters: *jFlag* and *jSlotNo* before sending data as follows:

(14) DATA=(r,P(r),jFlag,jSlotNo,payload)

An orphan node, say *x*, that overhears *DATA* judges the quality of link (*x*, *r*), uses *jFlag* to determine whether it is possible to join node *r*, and if possible, sends a join message using an unscheduled slot, *jSlotNo*, to node *r* to avoid collision. In this case, *jFlag* = 1 indicates that node *r* can have a new child, and *jSlotNo* is an unscheduled slot number in the frame that node *r* uses for slot scheduling. The joining process of an *Orphan* node is very similar to the node registration process, except that it uses a collision avoidance technique and has to explore a channel or group to join because they do not use a common channel.

Considering that there are multiple groups using different channels, an *Orphan* node shuffles *m* channels to have a channel explore list (*ChXpList*) and tries to overhear *DATA* to find a *1HopR* node with good link quality and *jFlag* = 1 during *k* frame periods, sequentially selecting a channel from *ChXpList* every UL frame period. Note that *ChXpList* helps distribute *Orphan* nodes evenly into different groups. An *Orphan* node also receives DL messages using a common channel at the beginning of every frame period for the same *k* frame periods. An *Orphan* node, *z*, after receiving DL messages, calculates link quality and decides whether or not it can become a *1Hop* node. If node *z* can become *1Hop* or *1HopR* node, it transmits *RR* including *uPF*(*z*) to GW using a randomly selected channel and waits for *uSSI*(*z*) in the next DL message. Otherwise, node *z* checks to see if it can be a *2Hop* node based on the *DATA* that it has overheard. Based on the overheard *DATA*, it selects an *1HopR* node with the best link quality and *jFlag* = 1, and joins the selected *1HopR* node by sending *RR* using *jSlotNo* on the channel that the *1HopR* node uses. Upon receiving *RR*, *1HopR* node *x* updates CS(*x*) = CS(*x*) ∪ {*z*}, generates *uPF*(*x*), and sends *RR* = (*x*, *P*(*x*), *uPF*(*x*)) to GW. Then, a server registers a new node *z*, generates *uSSI*(*x*), and broadcasts the DL message, including *uSSI*(*x*).

#### 3.5.3. Slot Scheduling Information Management

Given a list of *k* 1-hop nodes for group *i* as (*n_i1_*, *n_i2_*, …, *n_ik_*), a server maintains a network information table, *NIT*(*i*), as follows:

(15) $$NIT(i)=(n_{ij},TSD(n_{ij}),PF(n_{ij}),valid)$$

where valid indicates whether the corresponding entry is valid or not. Then, the start logical slot index *startLSI*(*n_ij_*) of node *n_ij_* in group *i* can be calculated easily by (5).

A server updates the table whenever it receives an updated profile from any 1-hop node during the data collection period. As mentioned in the subsection of problem identification, the update of the schedule can cause a slot schedule conflict problem. Two solutions to this problem can be considered. First, upon receiving *uPF*(*x*) from a *1HopR* node *x*, a server can produce *uSSI*(*x*) using a list of slots that do not include the slots allocated previously to node *x*. In this case, even though any child, say *y*, disconnected from node *x* sends data continuously, its transmission slot will never conflict with the new slot schedule of node *x*. As another method, upon receiving *uPF*(*x*), a server includes *Removed*(*y*) in the next DL message to indicate that node *x* has removed its child *y*. If node *y* receives it, fortunately, it changes its state to *Orphan* and attempts to join GW or any of relay nodes. In this case, the server may have to broadcast *Removed*(*y*) continuously until it receives *uPF*(*y*) or *uPF*(*P*(*y*)) from node *y* or node *P*(*y*) (if node *y* found a new parent), respectively. Upon receiving *uPF*(*y*) or *uPF*(*P*(*y*)), the server generates and broadcasts *uSSI*(*x*) for *y*’s previous parent *x*. This implies that the new scheduling of node *x* waits until node *y* finds its new parent, GW or *P*(*y*). The first method may not work well unless node *x* finds a sufficient number of unscheduled slots except for the slots allocated to itself previously. If this happens, it may have to migrate to another group. In the second method, if node *y* does not receive the DL message, it may have to wait long by broadcasting the *Removed(y)* continuously. One solution is to let node *y* change its type to *Orphan* if it misses DL messages for the specified number of frame periods.

In this paper, the first solution was implemented as follows. A *1HopR* node reports an updated profile to a server if it has a new child joined or loses any of its children, and then turns to a virtual node immediately. Upon receiving the updated profile, the server sets the validity of the corresponding entry to zero and changes the node to a virtual node (*v*) immediately in the *NIT*. Then, the *1HopR* node remains a virtual node, waiting for a new slot scheduling information from the server.

Suppose that a server receives *RR* = (*n_ij_*, *P*(*n_ij_*), *uPF*(*n_ij_*)) from node *n_ij_*. The server changes node *n_ij_* to a virtual node in *NIT*(*i)* and schedules *TSD*(*n_ij_*) either using the slots occupied by the other virtual nodes that have a sufficient number of slots or starting with *nextLSI*(*i*) as follows:

(16) $$nextLSI(i)=\sum_{x\in G{\left(i\right)}^{+}}TSD(x)+1$$

where $G{\left(i\right)}^{+}$ is a set of all nodes and virtual nodes that belong to group *i*.

For example, consider Table 1 in which *NIT*(*i*) has one virtual node *v*_1_. Suppose that *n_i2_* has reported *uPF(n_i2_)* with *TSD*(*n_i2_*) = 3 after losing one child. Then, the server allocates *TSD*(*n_i2_*) to a new virtual node *v*_2_ immediately. Then, node *n_i2_* obtains 3 slots from the slots occupied by *v*_1_, instead of its previous slots that are now occupied by *v*_2_. Then, the remaining 2 slots out of 5 slots are given to a new virtual node *v*_3_. A server broadcasts *uSSI*(*n_i2_*) as follows:

(17) $$uSSI(n_{i2})=(i,startLSI(n_{i2}),uPF(n_{i2}))$$

## 4. Evaluation

### 4.1. Simulation

#### 4.1.1. Channel Model

The simulation uses the log-distance path loss model with shadowing [25], which is widely used to model wireless channels in built-up and densely populated areas. Using this model, the path loss at communication distance *d* is described as follows:

(18) $$PL(d)=PL(d_{0})+10\gamma \log_{10}\frac{d}{d_{0}}+X_{\sigma }$$

where *PL*(*d*_0_) is the path loss at reference distance *d*_0_, γ is the pass loss exponent, *d* is the transmission distance (*d* > *d*_0_), and $X_{\sigma }$ is the zero-mean Gaussian distributed variable with standard deviation *σ*.

(19) $$p(X_{\sigma })=\frac{1}{\sigma \sqrt{2\pi }}\exp(-\frac{X_{\sigma }{}^{2}}{2\sigma^{2}})$$

The received power at distance *d*, $P_{r}(d)$, is calculated based on transmission power *P_t_* and path loss at distance *d* as follows:

(20) $$P_{r}(d)=P_{t}-PL(d)$$

Assuming that the LoRa signal can be demodulated if the received power is greater than or equal to receiving sensitivity of the receiver, $P_{\min}$. The probability of receiving a packet at distance *d*, $p_{receiving}(d)$, can be calculated as follows:

(21) $$p_{receiving}(d)=p(P_{r}(d)\geq P_{\min})$$

Refer to Section 2.7.2 in [25]:

(22) $$p_{receiving}(d)=Q(\frac{P_{\min}-P_{r}(d)}{\sigma })$$

where the *Q* function is defined as the probability that a Gaussian random variable *x* with mean zero variance one is greater than *z*:

(23) Q(z)=p(x>z)=∫z∞12πe−y22dy

#### 4.1.2. Simulation Setup

**Figure 8:**
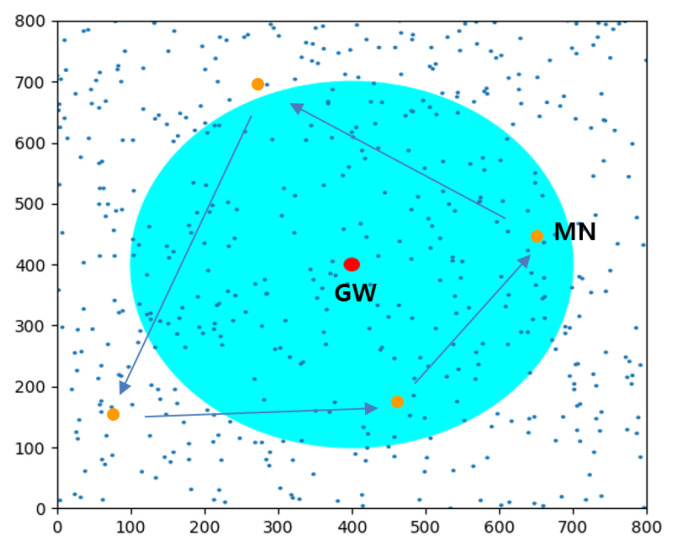
Example of spatial distribution with 500 end nodes in an area of 800 × 800 (m^2^).

For simulation, 500 nodes and 20 additional mobile nodes are randomly distributed in a square area of 800 × 800 m^2^, and one gateway denoted by a red circle is placed at the center, as illustrated in Figure 8. The big cyan-colored circle indicates the transmission range of GW and the small yellow-circle shows one example of a mobile node that moves along the arrows.

The two-hop LoRa network is constructed under the assumption that the connectivity between two nodes exists only if the link provides a receiving probability greater than 95%. For the values of the channel parameters, refer to the experimental results in [26] where *d*_0_ = 1 m, *PL(d_0_)* = 40.7 dB, *γ* = 3.54, and *σ* = 5.34. Every mobile node moves arbitrarily at the speed of 2 m/s that models the movement of workers carrying sensor nodes, and takes the pausing time that follows a Poisson process with an expected pausing time λ in minutes. The key parameters and values are listed in Table 2.

#### 4.1.3. Simulation Results

Some performance evaluation metrics are used as follows. The *packet delivery rate* (*PDR*) is the ratio of the number of packets received successfully by GW to the number of packets generated by all end nodes during simulation. Additionally, it may be meaningful to evaluate the quality of links. Thus, the packet delivery rate for the nodes except orphan nodes is evaluated under the premise that *Orphan* nodes do not transmit data, referred to as *PDR_NoOrphan* that is defined as the ratio of the number of packets received successfully at GW to the number of packets transmitted by all end nodes.

**Figure 9:**
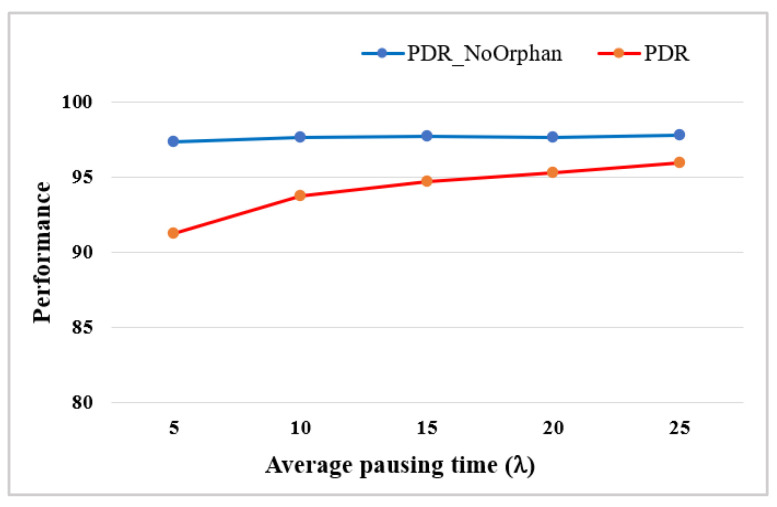
The average *PDR* and *PDR_NoOrphan* with different λ.

Simulations were performed over a period of 10,000 frames (=132,000 s), changing the value of λ from 5 to 25 min. Figure 9 shows the average *PDR* and *PDR_NoOrphan* of 20 mobile nodes. It is seen that the average *PDR_NoOrphan* remains high above 97% for all values of λ, while the average *PDR* increases from 91.2 to 95.9% as the value of λ increases from 5 to 25. The gap between two graphs implies that packet loss caused by the transmission of orphan nodes cannot be ignored.

**Figure 10:**
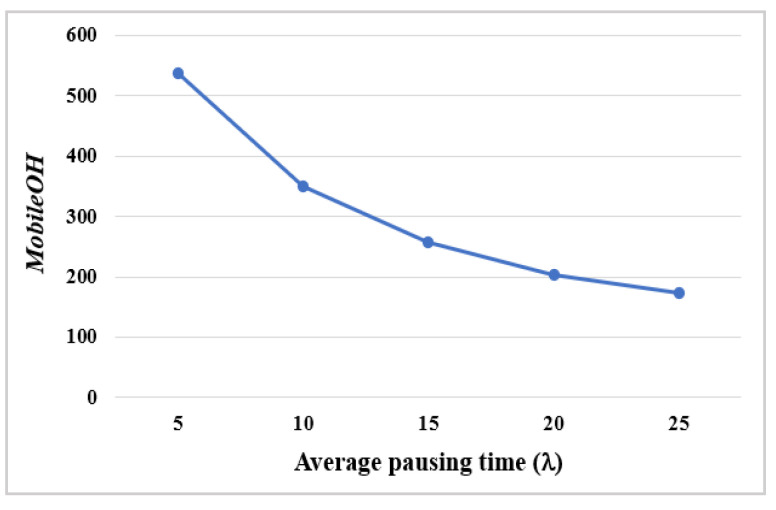
The average *MobileOH* with different λ.

Additionally, control overheads of the mobile node (*MobileOH*), which is defined as the number of control packets transmitted by the mobile node and its parent, was measured during simulation. Figure 10 shows the average *MobileOH* of 20 mobile nodes for different λ values. It is shown that *MobileOH* decreases sharply as λ increases up to 25. When λ = 5, the mobile node needs more than 500 control packets during simulation, which is 5% of the number of packets generated.

### 4.2. Experiment

#### 4.2.1. Experiment I with Static Node Scenario

**Figure 11:**
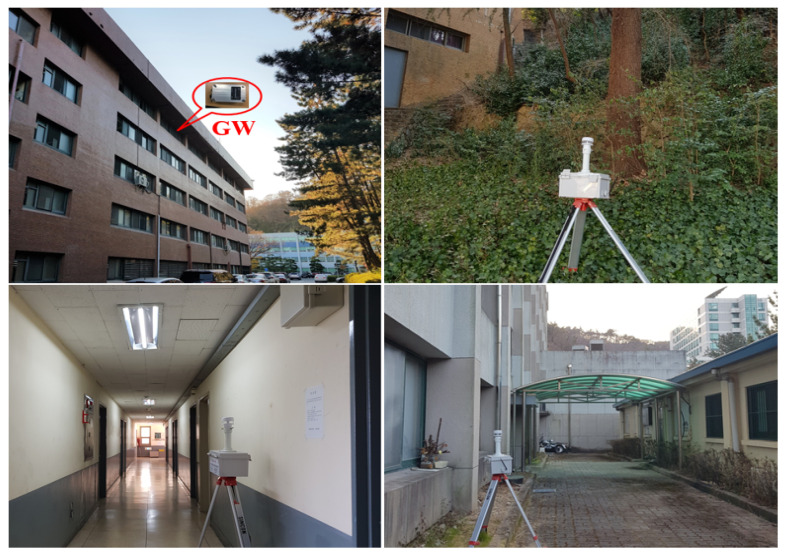
Photos of GW and some nodes installed in a university campus.

For the experiment, GW and node (in fact, weather device with an ultra-sound wind detector and a LoRa end node) were developed as shown in Figure 11. The GW consists of Raspberry PI 3 Model B+ and SX1301 LoRa transceiver, and the LoRa node consist of STM32L151xx and SX1276 LoRa transceiver. In this static node scenario, 1 GW and 40 nodes were deployed on a 500 × 350 (m^2^) area of a university campus. Each node receives GPS data, wind speed, wind direction, temperature and humidity from the weather detection system and sends those data to the GW periodically. Since some nodes were installed in communication shadow areas such as valleys on campus, inside buildings, and behind buildings, many nodes could not be directly connected to the GW. As shown in the upper left photo of Figure 11, the GW was installed in the lecture room on the 6^th^ floor of the computer science building, with the antenna exposed to the outside. The key experiment parameters and values are summarized in Table 3.

**Figure 12:**
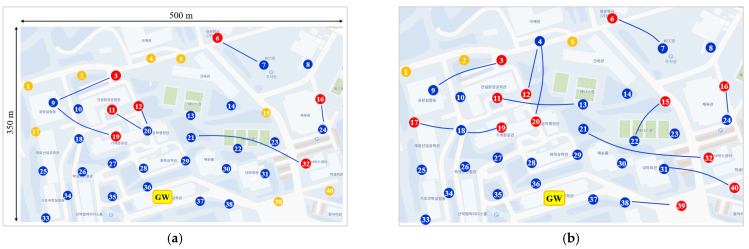

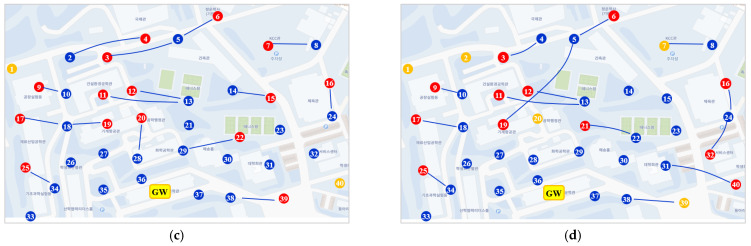
Static node deployment scenario and the change of topology. (**a**) Node connections after network initialization; (**b**) Node connections after 8 h of operation; (**c**) Node connections after 16 h of operation; (**d**) Node connections after 24 h of operation.

Figure 12a shows the two-hop topology constructed with (*RSSI_Th1*, *SNR_Th1*) = (−110 dBm, −3.5 dB) and (*RSSI_Th2*, *SNR_Th2*) = (−115 dBm, −5.5 dB) immediately after network construction. The blue colored, brown-colored, and red-colored circles indicate *1HopR*, *1Hop*, and *2Hop* nodes, respectively.

The experiment was conducted for several days, recording network connection status every 8 h as shown in Figure 12b–d. As shown in the figures, nodes 4, 5, 7, 15, and 20 change connections frequently due to their link instability. The reason is that node 20 was placed low behind the Chemical Engineering Building that blocks the building having the GW, and nodes 4, 5, and 15 were placed quite far from the GW and intercepted by several buildings and trees. Node 7 was placed in an open area but blocked by the hills because it is placed low. Nodes 20 and 7 started out as *1HopR* nodes and turned into *2Hop* nodes after 8 and 16 h, respectively, and then node 20 turned into *1Hop* nodes after 24 h.

Table 4 summarizes the number of 1-hop nodes, the number of 2-hop nodes, and the lowest and average *PDR_NoOrphan* values among the *PDR_NoOrphan*s of 1-hop and 2-hop nodes. The proposed protocol achieves a high *PDR_NoOrphan* of over 99% on average for 1-hop nodes and over 97% for 2-hop nodes. Node 15 had a low *PDR_NoOrphan* of 92.2% because its one-hop connection was unstable during the first 8 h. However, it can be seen that the *PDR_NoOrphan* continued to increase after node 15 became a 2-hop node or a 1-hop node with a stable connection. A similar situation occurred on node 7. After 8 h, node 7 connected to node 8 to become a *2Hop* node. After 24 h, it is shown that all 1-hop or 2-hop nodes could achieve a high *PDR_NoOrphan* of 95% or more.

#### 4.2.2. Experiment II with Mobile Node Scenario

**Figure 13:**
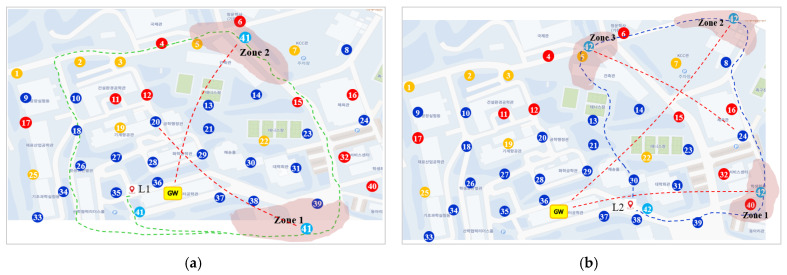
The change of connections according to node movement (The red-colored dashed lines indicate the unreliable connection). (**a**) Moving path of node 41; (**b**) Moving path of node 42.

In this experiment, two additional mobile nodes, 41 and 42, were allowed to travel along their respective predetermined paths over the previous static node scenario. At the starting point of the experiment, node 41 is installed inside the vehicle and travels along a green curved path starting at position L1 at a speed of 6 km/h (≈1.7 m/s) as shown in Figure 13a. After about 30 min, one of our lab members carried node 42 and moved from the starting point L2 along the blue curved path at about 4 km/h (≈1.1 m/s), which corresponds to the normal walking speed, as shown in Figure 13b.

Two mobile nodes moving along their respective paths performed data transmission while repeating the process of joining and leaving the already established and operating fixed LoRa network as in Experiment I. Mobile nodes, 41 and 42, move 4 laps and 1 lap, respectively, along their respective paths.

Table 5 summarizes the total experiment time, number of parent changes, *PDR_NoOrphan*, and *PDR* for two mobile nodes. It is shown that two mobile nodes achieve a high *PDR_NoOrphan* of over 90%. Taking advantage of the augmented transmission effect, the *PDR_NoOrphan* is improved to over 95%, the value in parentheses. This means that mobile nodes can transmit data quite reliably, even though they are constantly on the move. Note that two mobile nodes take a large difference of nearly 10% in PDR. The reason for this is that the slowly moving node 42 takes link disconnection time longer than mobile node 41.

Referring to Figure 13a, let us explain how mobile node 41 changes its node type. Starting as a *1Hop* type at point L1, mobile node 41 maintains a *1Hop* node during its first and second laps even though it has experienced link instability temporarily during these laps. However, in the third lap, when node 41 enters zone 2 pass zone 1 and its link becomes unstable, it became an orphan since it failed to receive the DL message three times in a row. Then, it gets node 20 as a new parent to be a *2Hop* type. In the fourth lap, when node 41 enters zone 1, it keeps the link to its parent despite that it is unstable temporarily. Then, it remains a *2Hop* type by the end of experiment.

Take a look at Figure 13b in which node 42 takes only one lap at a walking speed. Node 42 starts as an *1Hop* type at point L2. When it enters zone 1, its link becomes unstable, but remains same. Upon entering zone 2, node 42 changed its parent to node 24 since it failed to receive the DL message three times in a row. However, when it enters zone 3, it changes its parent back to GW due to its link instability. It is natural that node 42 changes its parent more since it maintains the state of link disconnection longer by moving more slowly than node 41.

## 5. Conclusions

A new multi-hop LoRa protocol for dynamic LoRa networks was presented by extending the previously proposed two-hop real-time LoRa protocol. It addressed the technical aspects of dynamic multi-hop networks, such as automatic configuration of multi-hop LoRa networks, dynamic topology management, and updating of real-time slot schedules and dealt with some technical issues related to node mobility. By resorting to both simulation and experiment, it was verified that the proposed protocol could achieve high reliability against signal attenuation and node mobility, but with low control overhead in topology management and schedule updates. According to experimental results with university campus deployment scenarios with forty nodes, the proposed protocol could achieve packet delivery rates of over 95% against node mobility.

## Figures and Tables

**Table 1:** Network information table, NIT(*i*).

(a) Before the Update of *n_i2_*				(b) After the Update of *n_i2_*			
*1Hop*	*TSD*	*PF*	Valid	*1Hop*	*TSD*	*PF*	Valid
*n_i1_*	3	*PF(n_i1_)*	1	*n_i1_*	3	*PF(n_i1_)*	1
*n_i2_*	5	*PF(n_i2_)*	1	*n_i2_* → *v*_2_	5	*-*	0
*…*				*…*			
*n_i(j−1)_*	1	*PF(n_i(j−1)_)*	1	*n_i(j−1)_*	1	*PF(n_i(j−1)_)*	1
*n_ij_* → *v*_1_	5	*-*	0	*n_ij_* → *n_i2_*	3	*PF(n_i2_)*	1
				*n_ij_* → *v*_3_	2	*-*	0
*n_i(j + 1)_*	1	*PF(n_i(j + 1)_)*		*n_i(j + 1)_*	1	*PF(n_i(j + 1)_)*	
*…*				*…*			
*n_ik_*	1	*PF(n_ik_)*	1	*n_ik_*	1	*PF(n_ik_)*	1
*…*				*…*			

**Table 2:** Simulation parameters.

Parameter	Value	Parameter	Value
No. GWs	1	Data rate (SF, BW)	7, 125
No. static nodes	500	No. UL slots	128
No. mobile nodes	20	UL slot size	100 (ms)
UL payload size	50 (bytes)	DL slot size	200 (ms)
Data transmission interval	13.2 (s)	Frame size	13.2 (s)
Expected pausing time (λ)	5, 10, 15, 20, 25	No. Data channels	8

**Table 3:** Experiment parameters and values.

Parameter	Value	Parameter	Value
UL slot size	100 ms	Data rate (SF, BW)	7, 125
DL slot size	200 ms	Frame Size	13.2 s
No. UL slot	128	Data transmission interval	13.2 s
No. UL channel	1	Payload size	24 bytes

**Table 4:** Experiment results.

	After Initialization	After 8 h	After 16 h	After 24 h
No. 1-hop nodes	33	28	25	26
No. 2-hop nodes	7	12	15	14
AVG. *PDR_NoOrphan* (1-hop)	N.A.	99.4%	99.4%	99.2%
Worst *PDR_NoOrphan* (1-hop)	N.A.	97.9%	97.7%	95.9%
AVG. *PDR_NoOrphan* (2-hop)	N.A.	97.5%	97.7%	98.5%
Worst *PDR_NoOrphan* (2-hop)	N.A.	92.2%	93.4%	95.2%

**Table 5:** Experiment result.

Node ID	41	42
Experiment time	1 h	25 min
Number of parent changes	1	2
*PDR_NoOrphan*	93.3 (97.4)	90.2 (96.5)
*PDR*	90.3 (94.3)	80.8 (86.4)
